# Mechanical Thrombectomy for Iliofemoral Deep Venous Thrombosis Complicated by Phlegmasia Cerulea Dolens in a Pregnant Patient With May-Thurner Syndrome: A Case Report

**DOI:** 10.7759/cureus.86570

**Published:** 2025-06-22

**Authors:** Jose Carlos Serrano Reyes, Rolando Pinilla, Gilberto Chanis, Rolando Jaen, Jose Valdes

**Affiliations:** 1 General Medicine, Paitilla Hospital, University of Panama, Panama, PAN; 2 Critical Obstetrics, Gynecology and Obstetrics, Paitilla Hospital, University of Panama, Panama, PAN; 3 Interventional Radiology, Paitilla Hospital, University of Panama, Panama, PAN; 4 Internal Medicine, Paitilla Hospital, University of Panama, Panama, PAN; 5 Emergency Medicine, Paitilla Hospital, University of Panama, Panama, PAN

**Keywords:** deep venous thrombosis (dvt), may-thurner syndrome, mechanical thrombectomy (mt), phlegmasia cerulea dolens, pregnancy

## Abstract

This case report describes a 35-year-old, G1P0 patient at 24 weeks and three days of gestation with no significant medical history, who presented with edema and discoloration of the left lower extremity, accompanied by left inguinal pain. These symptoms followed three days of low back pain radiating to the same region. On physical examination, findings were suggestive of deep venous thrombosis (DVT). Point-of-care ultrasound and venous Doppler confirmed thrombosis extending from the left proximal femoral vein to the left external iliac vein. To further assess the thrombus burden and anatomical extent, a computed tomography (CT) venography was performed following informed consent. CT venography demonstrated a thrombus extending from the left external iliac vein through the left common iliac vein to its junction with the inferior vena cava (IVC). The left common iliac vein was notably compressed by the overlying right common iliac artery, causing retrograde venous congestion and supporting the diagnosis of iliofemoral DVT secondary to May-Thurner syndrome. After thorough multidisciplinary discussion and risk-benefit evaluation, and with the patient's informed consent, an interventional approach was undertaken. Initial management included anticoagulation with enoxaparin, partial mechanical thrombectomy, and placement of an IVC filter. On the second day post-procedure, the patient developed acute worsening of edema, progressive skin discoloration, decreased temperature of the affected limb, and diminished palpable pulses, raising concern for arterial compromise consistent with phlegmasia cerulea dolens. In response, an extensive mechanical thrombectomy was promptly performed, resulting in improved distal venous outflow and clinical stabilization. This case demonstrates that, in selected pregnant patients with extensive or complicated iliofemoral DVT, mechanical thrombectomy within a multidisciplinary care framework can be a safe and effective therapeutic option to achieve rapid symptom relief and potentially reduce short- and long-term complications.

## Introduction

Pregnancy is recognized as a hypercoagulable state due to a multifactorial interplay involving increased levels of circulating clotting factors, reduced fibrinolytic activity, venous stasis due to decreased venous return, and direct mechanical compression of pelvic vessels by the gravid uterus [1-2]. As a result, pregnant individuals are at increased risk of developing deep venous thrombosis (DVT), leading to a substantial threat to maternal health due to associated complications and potentially compromising fetal development [3]. May-Thurner syndrome is a vascular condition in which the right common iliac artery compresses the left common iliac vein against the spine, predisposing to left-sided iliofemoral DVT. During pregnancy, this risk is amplified by additional extrinsic compression from the enlarging uterus and the inherent hypercoagulable state associated with pregnancy, significantly increasing the likelihood of progression from simple venous compression to clinically significant thrombosis. Phlegmasia cerulea dolens is a rare but severe complication of extensive acute iliofemoral DVT, marked by arterial compromise of the limb secondary to near-total venous occlusion of both deep and collateral veins. With near-total obstruction of the main venous outflow of the leg, there is virtually no venous return, which worsens congestion further. As venous congestion progresses due to fluid sequestration in the leg, interstitial and compartment pressures rise significantly. This increase in interstitial and compartment pressures can exceed the critical closing pressure of the arteries, resulting in the collapse of the arterial vessels. This collapse results in impaired arterial inflow, leading to tissue ischemia and, if untreated, potential gangrene. The progression from venous congestion to arterial collapse underscores the urgency of prompt diagnosis and intervention to prevent irreversible limb damage and systemic complications. The standard of care for thrombosis during pregnancy is anticoagulation, with low-molecular-weight heparin (LMWH) being the preferred agent due to its established safety profile and lack of transplacental transfer [4-6]. However, in select cases involving extensive thrombosis, rapid clinical deterioration, or contraindications to conventional therapy, interventional approaches such as catheter-directed thrombolysis (CDT) or mechanical thrombectomy have gained increasing attention [7-8]. These interventions aim to restore venous patency, alleviate symptoms more promptly, and reduce both short-term and long-term complications, such as post-thrombotic syndrome (PTS) [9-11]. PTS arises when the main outflow vein of the leg is obstructed, leading to valvular destruction and venous hypertension. This condition can result in persistent pain, swelling, skin discoloration, slow-healing venous ulcers, reduced mobility, and psychological impact. The cumulative effect of these symptoms can significantly impair a patient's quality of life, making early intervention and management crucial. Given the unique safety considerations during pregnancy, the role of such advanced therapies continues to evolve, warranting further clinical investigation in this specific population.

## Case presentation

A 35-year-old pregnant woman, gravida 1 para 0, at 24 weeks and three days of gestation, with no significant past medical history, presented to the emergency department with a 24-hour history of swelling and discoloration of the left lower extremity, accompanied by localized pain in the left inguinal region. She reported the onset of lower back pain three days prior, which had progressively radiated toward the inguinal area. At presentation, the patient was hemodynamically stable, with no signs of respiratory distress.

Physical examination revealed diffuse edema of the entire left lower limb, cutaneous discoloration, and visible superficial collateral venous circulation. Localized tenderness was noted in the left inguinal region. Distal pulses were intact, with preserved neurovascular function, normal skin temperature, and capillary refill time under two seconds.

**Figure 1:**
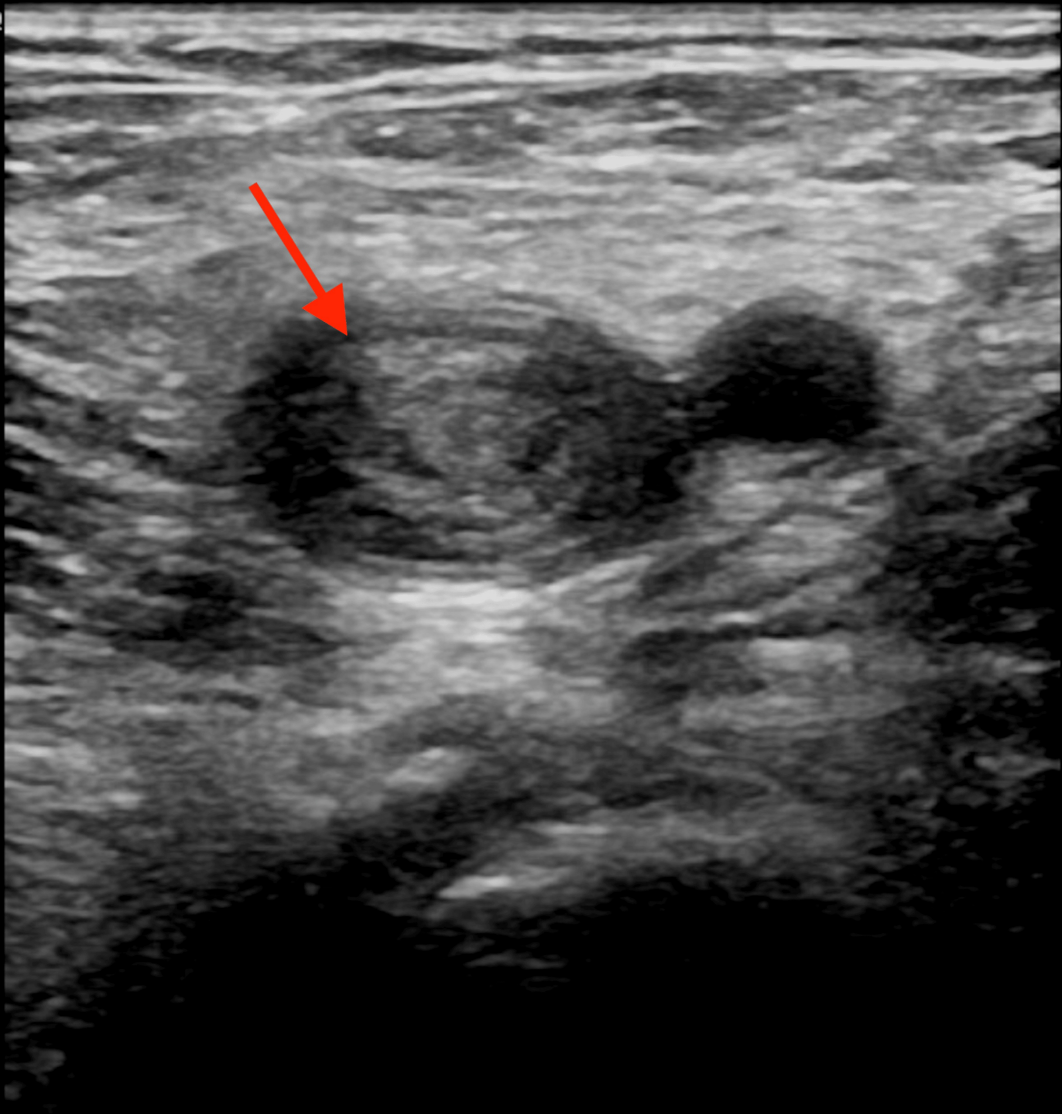
Compression ultrasound demonstrating a non-compressible, hyperechoic thrombus (red arrow) occupying the entire lumen of the left common femoral vein

A point-of-care ultrasound compression ultrasound demonstrated a non-compressible, hyperechoic thrombus occupying the entire lumen of the proximal left femoral vein, consistent with DVT (Figure 1). The thrombus extended proximally to the visualized portion of the external iliac vein. Venous Doppler ultrasound confirmed these findings, and further imaging was recommended to assess the possible involvement of the left common iliac vein and IVC.

**Figure 2:**
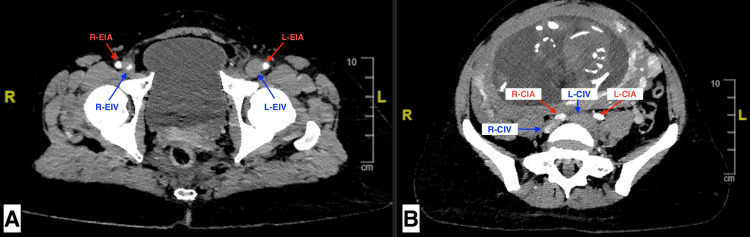
CT venography showing thrombus extension from the left external iliac vein to the left common iliac vein A. Filling defect and dilation of the left external iliac vein (L-EIV) compared to the right external iliac vein (R-EIV). B. Filling defect and dilation of the left common iliac vein (L-CIV) compared to the right common iliac vein (R-CIV), due to extrinsic compression by the right common iliac artery (R-CIA) and the gravid uterus. CT: computed tomography

To further assess the thrombus burden and anatomical extent, a low-dose contrast-enhanced CT venography of the abdomen and pelvis was performed following informed consent by the patient. The CT venography revealed a filling defect in the left common femoral vein, along with dilation of the external and common iliac veins, extending to the level of extrinsic compression by the right common iliac artery and gravid uterus (Figure 2). These findings on the CT scan confirmed the presence of May-Thurner syndrome.

**Figure 3:**
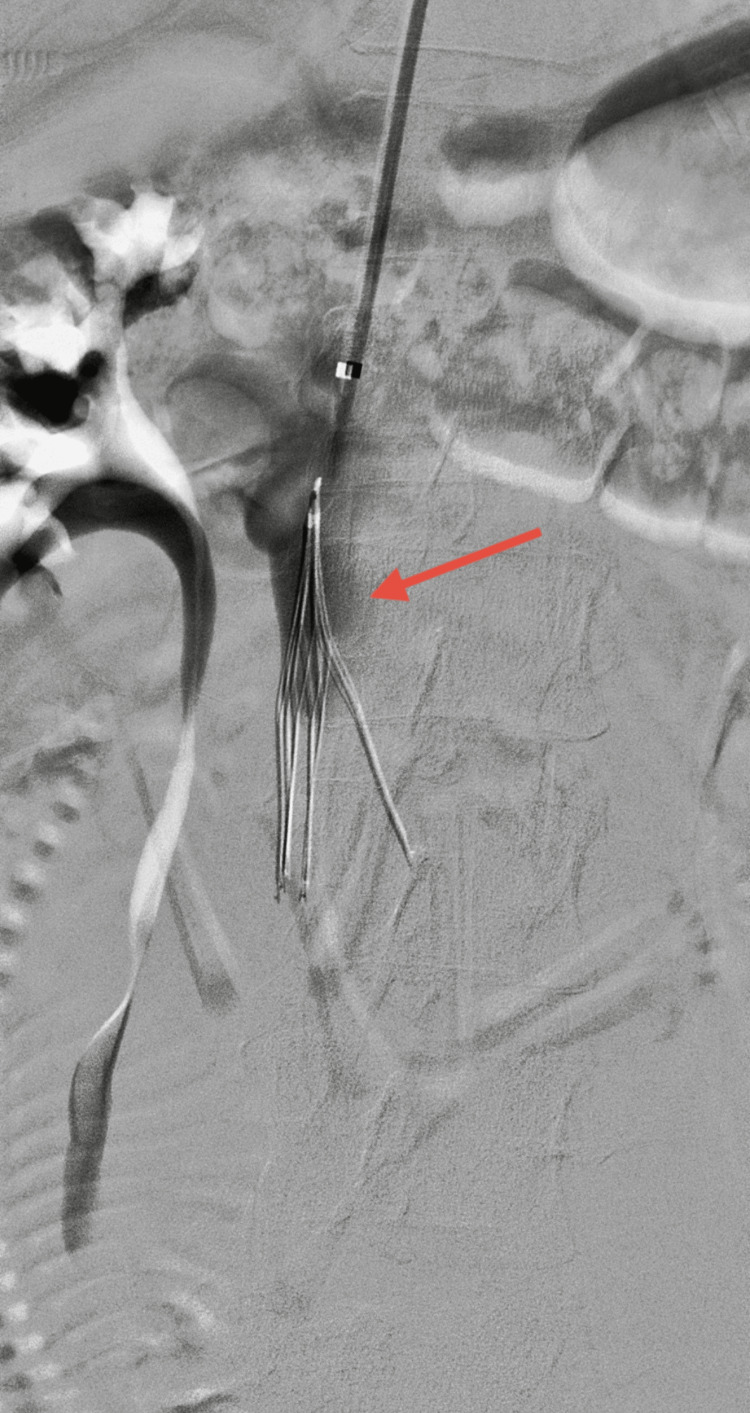
Placement of an IVC filter (red arrow) to prevent pulmonary embolism IVC: inferior vena cava

Initial management involved therapeutic anticoagulation with LMWH (enoxaparin, 1 mg/kg) and multidisciplinary consultation with obstetrics, maternal-fetal medicine, and interventional radiology. Following a thorough review and with the patient’s informed consent obtained after multidisciplinary evaluation, a partial mechanical thrombectomy was performed under low-dose fluoroscopy to minimize fetal radiation exposure. In addition, a prophylactic IVC filter was placed to reduce the risk of pulmonary embolism (Figure 3).

**Figure 4:**
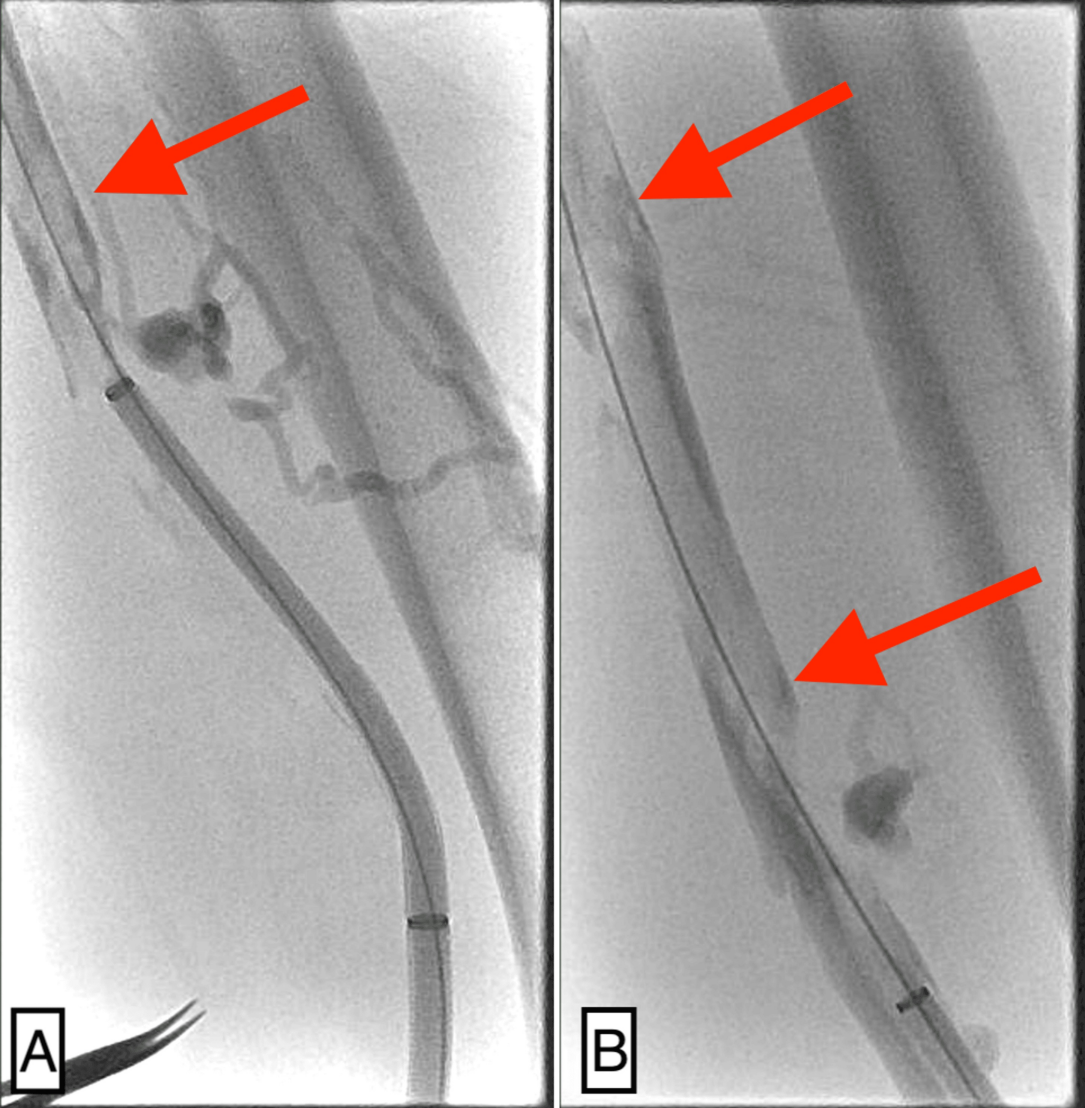
Venogram demonstrating thrombus in the femoral vein (red arrows) A. Venogram showing a thrombus in the femoral vein (red arrow) with clear visualization of the catheter and guidewire of the thrombectomy system. B. Extended thrombus (red arrows) involving a more proximal segment of the femoral vein.

**Figure 5:**
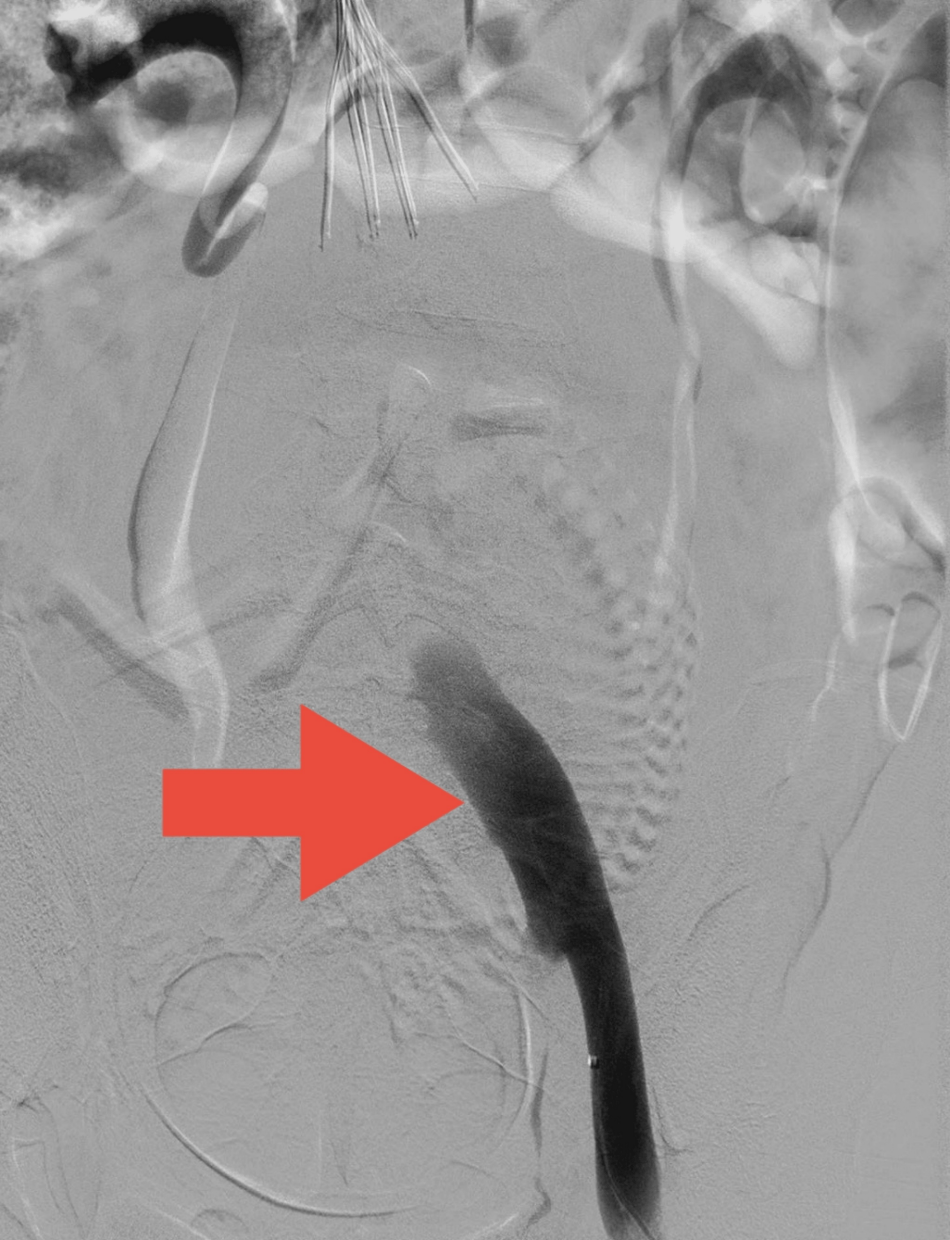
Post-treatment venogram demonstrating flow through the common iliac vein (red arrow)

On postoperative day two, the patient experienced worsening edema, increased discoloration, distal cyanosis, decreased temperature, and diminished palpable pulses of the affected limb. These clinical signs were highly suggestive of recurrent DVT with potential arterial compromise, consistent with phlegmasia cerulea dolens. In response, after obtaining informed consent, an extensive mechanical thrombectomy was performed using the INARI ClotTriever System (Figure 4), which resulted in significant improvement in venous drainage (Figure 5) and symptomatic relief. The total radiation exposure was 486 mGy, which is within the range generally considered safe for a fetus in the second and third trimesters of pregnancy [12].

Postoperatively, the patient was maintained on enoxaparin 1 mg/kg subcutaneously every 12 hours. Her symptoms improved significantly, and she was discharged on postoperative day three in stable condition. The remainder of her pregnancy proceeded without further complications.

## Discussion

The management of DVT during pregnancy presents unique challenges due to the need to carefully balance maternal and fetal risks. LMWH remains the first-line pharmacological treatment, as broadly recommended by current clinical guidelines, due to its established safety profile during pregnancy [5-6].

In cases of extensive thrombotic burden or complications such as phlegmasia cerulea dolens, where arterial flow may be compromised, direct thrombus removal techniques, such as CDT and mechanical thrombectomy, have demonstrated high efficacy and are strongly recommended by current guidelines for use in the non-pregnant population [7]. These interventions provide rapid symptom relief and help prevent long-term complications such as PTS. Although both CDT and mechanical thrombectomy have been successfully used in selected pregnant patients [8-10], their application remains controversial due to limited evidence and potential risks, including maternal hemorrhage, placental disruption, preterm labor, and fetal compromise. In the absence of strong recommendations for use during pregnancy, these advanced therapies should be reserved for life-threatening cases involving a high thrombotic burden, particularly acute iliofemoral DVT, or when limb ischemia is imminent, as in phlegmasia cerulea dolens [6]. In such scenarios, the decision to proceed should be made within a multidisciplinary team, carefully considering both short- and long-term risks and benefits for the mother and the fetus.

Given the direct risk of maternal hemorrhage associated with pharmacological thrombolysis, we opted for mechanical thrombectomy as the preferred intervention in this case. Despite the inherent radiation exposure, mechanical thrombectomy was strategically employed with dose minimization protocols, allowing effective treatment without compromising fetal safety. This resulted in improved distal venous perfusion and favorable clinical outcomes, with no complications.

Mechanical thrombectomy has been used safely in a limited number of pregnant patients during the first and second trimesters [10,11]. However, although measures to reduce fluoroscopy time were implemented, prenatal radiation exposure continues to carry potential risks, especially during the first trimester of pregnancy, when the risk of fetal harm is most significant. An alternative approach, previously described in first-trimester cases, involves the use of intravascular ultrasound-guided mechanical thrombectomy without the need for fluoroscopy [13,14]. This technique further minimizes fetal radiation exposure, though visualization may become more difficult as pregnancy progresses due to uterine size. While further research is required to validate the safety and efficacy of these technologies in pregnant populations, the successful use of mechanical thrombectomy in the present case suggests it may represent a feasible alternative to anticoagulation alone in select high-risk scenarios.

## Conclusions

Mechanical thrombectomy proved to be a viable and well-tolerated approach for managing complicated iliofemoral DVT secondary to May-Thurner syndrome in a patient during the second trimester of pregnancy. While anticoagulation remains the standard therapy for pregnancy-associated DVT, selected cases may benefit from early thrombus extraction when rapid symptom control and prevention of life- or limb-threatening complications are critical therapeutic goals.

## References

[1] Waheed SM, Kudaravalli P, Hotwagner DT (2025). Deep vein thrombosis. StatPearls [Internet].

[2] Kumar DR, Hanlin E, Glurich I, Mazza JJ, Yale SH (2010). Virchow's contribution to the understanding of thrombosis and cellular biology. Clin Med Res.

[3] Stone J, Hangge P, Albadawi H (2017). Deep vein thrombosis: pathogenesis, diagnosis, and medical management. Cardiovasc Diagn Ther.

[4] Alquwaizani M, Buckley L, Adams C, Fanikos J (2013). Anticoagulants: a review of the pharmacology, dosing, and complications. Curr Emerg Hosp Med Rep.

[5] Ortel TL, Neumann I, Ageno W (2020). American Society of Hematology 2020 guidelines for management of venous thromboembolism: treatment of deep vein thrombosis and pulmonary embolism. Blood Adv.

[6] Bates SM, Greer IA, Middeldorp S, Veenstra DL, Prabulos AM, Vandvik PO (2012). VTE, thrombophilia, antithrombotic therapy, and pregnancy: antithrombotic therapy and prevention of thrombosis, 9th Ed: American College of Chest Physicians evidence-based clinical practice guidelines. Chest.

[7] Meissner MH, Gloviczki P, Comerota AJ (2012). Early thrombus removal strategies for acute deep venous thrombosis: clinical practice guidelines of the Society for Vascular Surgery and the American Venous Forum. J Vasc Surg.

[8] Devis P, Knuttinen MG (2017). Deep venous thrombosis in pregnancy: incidence, pathogenesis and endovascular management. Cardiovasc Diagn Ther.

[9] Du X, Zhuang H, Hong L (2019). Long-term outcome of catheter-directed thrombolysis in pregnancy-related venous thrombosis. Med Sci Monit.

[10] Pillny M, Sandmann W, Luther B (2003). Deep venous thrombosis during pregnancy and after delivery: indications for and results of thrombectomy. J Vasc Surg.

[11] Herrera S, Comerota AJ, Thakur S, Sunderji S, DiSalle R, Kazanjian SN, Assi Z (2014). Managing iliofemoral deep venous thrombosis of pregnancy with a strategy of thrombus removal is safe and avoids post-thrombotic morbidity. J Vasc Surg.

[12] Yoon I, Slesinger TL (2025). Radiation exposure in pregnancy. StatPearls [Internet].

[13] Ichinose EJ, Mouawad NJ (2023). Ultrasound-guided mechanical thrombectomy without radiation for deep vein thrombosis in early pregnancy. JACC Case Rep.

[14] Gedikoglu M, Oguzkurt L (2017). Endovascular treatment of iliofemoral deep vein thrombosis in pregnancy using US-guided percutaneous aspiration thrombectomy. Diagn Interv Radiol.

